# In Nature's School

**Published:** 1889-07-13

**Authors:** 


### THE DOCTOR'S PULPIT.

### IN NATURE'S SCHOOL.

### WILD ROSES.

IT is not alone the child bred in towns and taken to the country for a few days or weeks in the summer who feels disposed to clap his hands and exclaim "How beautiful!" when he sees a tall spreading bush, or a whole hedgerow covered with wild roses at every stage of "blowing" and in every shade of pink. The sight is one of the sweetest and simplest which Nature has to offer. And yet, in everything that constitutes perfection in the queen of flowers, the wild rose, with all its exquisite delicacy and beauty, is to the perfect rose of the garden "as moonlight is to sunlight, and as water is to wine." It is not possible for the writer even to hint any disparagement to the princess of the hedgerow. No one can be more loyal to every product of unaided Nature than he is. But truth is truth, and neither the scent nor the size, nor the colour nor the *tout ensemble* of the wild rose can compare with the finest products of the garden and the lawn.

Is, then, man greater and more powerful than Nature? By no means. The garden rose owes as much to Nature as her less cultured sister of the hedgerow. The wild rose seems to say to man, "See how pretty I am, even here in my native condition; take me under your care and protection, and I will become far more perfect still." The garden rose seems to continue the story and say, "I was wild and thorny and not very richly scented when you took me from the field; but I am now rich in perfume, noble in shape and size, glorious in colour, and the queen of the flowers. This is the reward you have for your labour and care." If we wish to compare the work of man alone with the work of Nature alone, we must not put into comparison the rose of the garden and the rose of the field. We must take the paper rose and the wax rose of the artificial flower maker and place them side by side with Nature's handiwork. There can be no doubt then whether Nature or man will carry the palm. What this little rose history shows is not that man is superior to Nature, but that Nature permits him to take some of her own products and, by care and labour, to develop them to a degree of perfection altogether unknown without such care. It is worth a consideration that man alone among all living and breathing creatures is able to do this.

It is only when man proceeds on what may be called "perfectly natural lines" that he can develop and improve Nature's productions. Many men, being both stupid and wilful, and finding that Nature may often be improved upon, begin to think themselves altogether superior to her. Instead of studying her, and working with her, they often set her at defiance, and pursue any course, be it natural or unnatural, which best pleases themselves. Those people very soon demonstrate their own folly both to themselves and to others. A rose will increase in size and improve in quality if it be placed in improved conditions of climate, scil, and care. But pluck it from the soil altogether, or fail to give it water and pure air, or remove it to a climate too cold for its liking, and it will soon show by its drooping petals that co-operation, and not contradiction, is what Nature asks and insists upon at the hands of man. All these circumstances have their analogies in the sphere of health. There is nothing more certain than that by proceeding on natural lines the worst health may be improved, and the feeblest constitution strengthened. Of course, for the very poorest, it is impossible to proceed always and altogether on natural lines, at any rate, in large towns. Some of the prime conditions of health are entirely wanting. Pure air, for example, cannot be had, nor even an adequate quantity of air such as there is. Food is often both bad and insufficient, whilst exercise is almost impossible. But the majority of people, if they had but sense to understand and strength of mind to carry out the first principles of health on natural lines, would find themselves in an altogether better condition than they now are. Health, like wealth, demands both effort in the winning and care and sense in the keeping. The careless man and the fool seldom have any large store either of the one or the other.

The wild rose and her imperial sister of the lawn suggest a defence of civilisation, and indicate the course it should take, and the limits which should bound it. Many people are enamoured of man in his natural condition. The greatest of civilised minds often feel a profound disgust with civilisation as it is. When Tennyson was jilted by Amy of the shallow heart, he threatened to "take some savage woman," to "rear his dusky race." And, undoubtedly, civilisation puts a great strain on men and women at every period of life except infancy and extreme old age. Naturalness with many people is entirely lost; naturalness, with the ease and freedom and friendliness which spring therefrom. Instead of it, we have a cold, suspicious, distrustful artificialism, with a constant sense of effort after something which is never reached.

But whatever be the wearisome toil which civilisation compels and the disgust to which it often gives rise, it is certain that, compared with the effortless, purposeless, and unintellectual life of the savage, it is happiness itself. Civilisation, with all the strenuous toil of brain and muscle which it implies, is the natural and inevitable result of high intellectual capacity. Given the brains of the Greeks, or the moral faculties of the Hebrews, and a permanent condition of unthinking and unheeding animalism was impossible. Man is made for and capable of a much higher civilisation than he has yet attained to, and the effort to reach a higher level is necessary and conducive to the most perfect health. Nature has supplied the wild rose of the untutored man. She has placed him in circumstances where development is possible; she has planted within him an unconquerable resolution to make the most both of his powers and his opportunities; and, following her lead steadily, moderately, and with "his eyes in his head," as Solomon says, man has become, and will become still more, the greatest of all Nature's great and mighty works. To eat is natural, to sleep is natural, to love is natural, to enjoy is natural; but just as natural as all these is hard thinking, severe self-denial for worthy objects; obedience to the highest law we know; and reverence for the greatest and noblest intellect we can discover. All moral worthiness is natural; and all moral worthiness is conducive to the most perfect health.

In such a world as ours, of opposed philosophies, conflicting religions, and personal strifes and struggles, it is impossible to imagine any intelligent man of forty who has not often asked himself in desperation: "Is there any single right way of living? are there any real laws of duty, which to obey is well-being, to disobey is injury and unhappiness? Is there any personal deity who guides the soul, or are there any ascertainable laws of Nature to which the intellect may conform itself?" In the judgment of the present writer there is a personal Deity who guides the docile spirit, and there are ascertainable laws of Nature to which the intellect may conform itself. Life conducted on these lines cannot but progress in the right direction; but, in any case, disregard of the clear and ascertainable laws of Nature is always and everywhere disastrous. Nature has made the human infant, like the wild rose of the field, capable of indefinite improvement by cultivation. The successive stages of a human being's life and experience may be likened to successive generations of roses whose original stock has been removed from the field to the garden. The great difference is that whilst the gardener must always cultivate the rose, even to the latest generation, man must be his own cultivator to a very large extent. In early childhood, as soon as intelligence opens and will develops, responsibility begins. When adult life is reached, every man and woman becomes his own gardener, and on his sense and behaviour depend the finished results of his life and experience. If he is a reasonable and an honest self-gardener, the rose of his life will be both fragrant and beautiful; if he be careless and worthless it may wither on its stem, and even its leaves shall exhale only odours that are disgusting and poisonous. Nothing is plainer than that earnest thought and honest purpose are twin guides to sound health, as well as to every other conceivable kind of excellence.

